# Too Much of a Good Thing: A Case of Acute Fluoride Ingestion

**DOI:** 10.7759/cureus.84221

**Published:** 2025-05-16

**Authors:** Swet Patel, Yevgeniy Polunin, James Espinosa, James Lee, Alan Lucerna

**Affiliations:** 1 Emergency Medicine, Jefferson Health New Jersey, Stratford, USA

**Keywords:** acute fluoride ingestion, emergency medicine management of acute fluoride ingestion, fluoride metabolism, management of acute fluoride ingestion, toxicity from acute fluoride ingestion

## Abstract

A protective effect of fluoride for the prevention of dental caries was observed in communities that consumed naturally fluoridated water compared to non-fluoridated water. Subsequently, fluoride began to be added to the drinking water in various countries as a means of caries prophylaxis. Dentistry began to develop the use of fluoride as an additive to toothpastes and mouth rinses as well as in the form of sodium fluoride powder. Fluoride gels, tablets, and powders can be used safely but can lead to acute ingestions. Here we present the case of a 36-year-old male patient with a history of depression and a previous salicylate overdose who presented to the emergency department (ED) with nausea, vomiting, and diffuse abdominal discomfort. He denied any prescription medications. On specific questioning concerning supplements obtained in a store or by the internet, he stated that he had ingested sodium fluoride powder. The patient said that he had been brushing with the powder for several days for dental pain. He stated that he ingested "a large amount" of the powder approximately eight hours prior to presentation at the ED. The powder had been purchased from the internet. He denied suicidal ideation. He stated that he brushed “a lot” in the eight hours prior to presentation at the ED and used half of the bottle of the powder. According to the patient and his family, the patient had an episode of hematemesis just prior to him presenting at the ED. The patient was found to have hypocalcemia and hypomagnesemia as well as hypotension and tachycardia. Gastroenterology performed an emergency esophagogastroduodenoscopy (EGD), which showed mild esophagitis and gastritis secondary to caustic injury. This case illustrates the importance of taking a complete medical history, including all non-prescription medications and supplements. It also illustrates that fluoride ingestion can present with tachycardia and hypotension as well as an elevated lactate, creating a sepsis-mimic picture. The importance of early repletion of electrolytes and consultation with gastroenterology is also illustrated by this case.

## Introduction

Fluoride is found on the earth's crust and is present in seawater and freshwater. Finite amounts of the element are found in mineralized tissues of the body such as enamel, dentin, and bone [1]. The drinking water content of fluoride is a function of the fluoride content of the crust of the local earth. A protective effect of fluoride on the prevention of dental caries was observed in communities that consumed naturally fluoridated water compared to non-fluoridated water. Subsequently, fluoride was added to the drinking water in various countries as a means of caries prophylaxis [2]. Dentistry began to develop the use of fluoride as an additive to toothpastes and mouth rinses as well as in the form of sodium fluoride powder. Fluoride has been described as a double-edged sword in that adequate amounts can be beneficial to dental health but excessive amounts can cause harmful effects on teeth and lead to a staining pattern known as fluorosis [1]. As noted by Guth et al., the risks and benefits of fluoride exposure in such various uses as drinking water supplementation and dental care product use have been heavily debated [2]. In addition, fluoride gels, tablets and powders can be used safely but can lead to acute ingestions [3].

This case report was presented in poster form at the Rowan University Research Day, Stratford, New Jersey, on May 7, 2023.

## Case presentation

A 36-year-old male patient with a history of depression, chronic dental hygiene issues, and previous salicylate overdose presented to the Emergency Department (ED) with nausea, vomiting, and diffuse abdominal discomfort. He denied any use of prescription medications. Asked about supplements, he stated that he had ingested sodium fluoride powder. The patient reported that he had been brushing with the powder for several days for dental pain. He denied suicidal ideation. He stated that he brushed “a lot” in the eight hours prior to presentation at the ED and used half bottle of the powder. The size and formulation of the fluoride bottle was not able to be determined. According to the patient and his family, he had an episode of blood-tinged hematemesis just prior to presenting at the ED. The bottle was not available on arrival. He denied fevers, chills, difficulty swallowing, chest pain, active melena, and hematochezia.

The patient was awake, alert, and oriented. The patient’s initial vital signs were as follows: blood pressure, 97/62 mmHg, temperature, 99.1 F, heart rate 117 beats per minute, respiratory rate 22 breaths per minute with an oxygen saturation of 100% on room air. The physical exam elicited diffuse abdominal tenderness with no peritoneal findings. The physical exam was otherwise unremarkable. No gross dental caries were noted.

In the ED, laboratory tests were ordered and the results are presented in Table 1.

**Table 1:** Results of laboratory investigations. BUN: blood urea nitrogen; PT: protime; PTT: partial thromboplastin time; INR: International Normalized Ratio; AST: aspartate aminotransferase; ALT: alanine aminotransferase; K/uL: 1000 per microliter; g/dL: grams per deciliter; mEq/L: milliequivalents per liter; mg/dL: milligrams per deciliter; mcg/ml: micrograms per milliliter; cells/HPF: cells per high-powered field; mmol/L: millimoles per liter. Values in bold are those below the normal levels.

Parameters	Result	Normal range	Units
White blood cell count	11.2	4.0-11.0	K/uL
Hemoglobin	11.3	10.6-15.6	g/dL
Platelet count	180.0	150-400	K/uL
Sodium	137.0	135-154	mEq/L
Potassium	3.6	3.5-5	mEq/L
BUN	18.0	5-20	mg/dL
Creatinine	1.0	0.6-1.2	mg/dL
Glucose	95.0	70-100	mg/dL
Calcium	7.3	8.5-10.5	mg/dL
Chloride	101.0	95-105	mEq/L
Bicarbonate	24.0	23-29	mEq/L
Magnesium	0.9	1.7-2.2	mg/dL
Lactate	4.1	0.5-2.2	mmol/L
PT	11.0	11-13.5	seconds
PTT	33.0	25-35	seconds
INR	1.0	0.8-1.1	INR ratio
Salicylates	Negative	Negative	mg/dL
Acetaminophen	Negative	Negative	mcg/ml
Urine drug screen	Negative	Negative	NA
Total bilirubin	0.8	0.1 to 1.2	mg/dL
AST	20.0	8 to 33	U/L
ALT	22.0	7 to 56	U/L
Urine color	Clear	Yellow	NA
Urine clarity	Clear	Clear	NA
Urine specific gravity	1.0	1.005-1.030	NA
Urine pH	7.0	5 to 7.5	NA
Urine glucose	Negative	Negative	NA
Urine protein	Negative	Negative	NA
Urine bilirubin	Negative	Negative	NA
Urine urobilinogen	Negative	Negative	NA
Urine ketones	Negative	Negative	NA
Urine blood	Negative	Negative	NA
Urine white cells	Negative	0-5/HPF	cells/HPF
Urine red cells	Negative	0-5/HPF	cells/HPF
Urine nitrite	Negative	Negative	NA
Urine leukocyte esterase	Negative	Negative	NA
Urine culture	No growth	No growth or <10K CFU	NA

The patient had hypomagnesemia and hypocalcemia. An elevated lactate level was noted. A chest x-ray showed no acute findings. An electrocardiogram showed sinus tachycardia but was otherwise unremarkable. An arterial blood gas was normal as was a urine drug screen. Poison control was called and repletion of the patient’s magnesium and calcium recommended and emergency esophagogastroduodenoscopy (EGD) ordered to rule out esophageal and gastric caustic injury. Gastroenterology performed an emergency EGD, which showed mild esophagitis and gastritis secondary to caustic injury.

Since the initial vital signs showed tachycardia and tachypnea, the patient received a bolus of 30 cc/kg saline as well as broad-spectrum sepsis antibiotics (vancomycin and piperacillin/tazobactam). The blood pressure improved to 130/70 mmHg. The patient was admitted to the hospital for an accidental fluoride overdose with a plan for serial abdominal exams, intravenous pantoprazole, and monitoring of the patient’s calcium and magnesium. Serial abdominal exams were unremarkable throughout the hospital stay. The lactate normalized over the next 24 hours. Calcium and magnesium were repleted and the hemoglobin remained in the normal range. Blood cultures were negative. The patient was discharged with plans to continue on oral proton pump inhibitors for eight weeks with follow-up outpatient appointments with gastroenterology and dentistry.

## Discussion

Most toxic exposures to fluoride are through the oral route and the most common compound is sodium fluoride, which is found in tooth powders and toothpaste [4]. Sodium fluoride is absorbed via the gastrointestinal tract and is excreted by the kidneys [5,6]. This powder is almost completely absorbed from the gastrointestinal tract into the blood with optimal plasma levels attained shortly after the oral ingestion of which the largest amount of absorbed fluoride is retained in bone and teeth [4]. Fluoride that is not incorporated into bone is renally excreted [7].

Acute fluoride ingestion can lead to irritation of the gastrointestinal tract causing abdominal pain, nausea, and vomiting. Diarrhea can occur. Fluid losses can cause hypotension. Ingested fluoride ion binds to circulating calcium and can cause severe hypocalcemia. Severe hypocalcemia can often lead to generalized or localized muscle tetany as well as seizure activity. Ingested fluoride can also bind to magnesium and can cause hypomagnesemia [8]. Delayed hyperkalemia can result. The mechanism of delayed hyperkalemia is not clear but may be due to potassium efflux from cells due to the effect of hypocalcemia on calcium-dependent potassium channels [7].

Fluoride toxicity must be evaluated immediately for the type and amount of ingested fluoride. The actual dose of fluoride ingested was not able to be calculated in this case. Bayless and Tinanoff point out that the difficulty in making such calculations can be related to the fact that there are a wide variety of fluoride preparations containing various concentrations and fluoride compounds (such as stannous fluoride, fluoride with sodium and sodium monophosphate). Fluoride can be obtained in gel, drops, tablets, and powders. A particular form may be available in several concentrations [6].

As with all patients, evaluation and management of airway, breathing, and circulation is fundamental. An emetic agent is contraindicated because of the danger of aspiration of gastric contents and burning of the esophagus due to hydrofluoric acid present in the stomach. A decision to perform gastric lavage can be made with poison control consultation and may be considered with a large-volume fluoride ingestion presenting within one hour of ingestion [4]. 

Hypotension can be due to vomiting and diarrhea and can be treated initially with fluid resuscitation, as was done in this case. Repletion of hypocalcemia and hypomagnesemia, which was present in this case, is a critical aspect of management. Endoscopy should be strongly considered, as was done in this case. If late hyperkalemia occurs, dialysis may be considered [7]. Tachycardia can be seen with significant fluoride ingestion, as was seen in the case presented. Some patients may develop ventricular arrhythmias, which is thought to be due to hypocalcemia and hypomagnesemia. Electrolyte repletion is crucial in such cases [9]. The fluoride ion, along with the electrolyte abnormalities, can cause metabolic acidosis [10]. This was not seen in our case.

Abukurah et al. reported a case of acute fluoride ingestion in which an esophageal stricture resulted [5]. McIvor reported a fatal case of delayed hyperkalemia and ventricular fibrillation after an acute fluoride ingestion [4]. Ventricular fibrillation after an acute fluoride ingestion was also reported by Chan and Duggin [10]. 

The lethal dose of ingested fluoride in adults has been proposed to be 32-64 mg of fluoride per kilogram of body weight. The lethal dose of ingested fluoride in children has been proposed to be 15 mg of fluoride per kilogram of body weight [11]. A crucial point is that acute fluoride toxicity from ingestion is preventable [3].

## Conclusions

Fluoride gels, tablets, and powders can be used safely but can lead to acute ingestions. Here we present the case of a 36-year-old male patient with a history of depression, chronic dental hygiene issues, and a previous salicylate overdose who presented to the ED after ingestion of an unknown amount of sodium fluoride powder. Fluoride ingestion can cause tachycardia and hypotension as well as an elevated lactate and can initially mimic a sepsis presentation. Esophagitis and more serious esophageal and gastric injury can be seen. Electrolyte imbalances, including significant hypocalcemia and hypomagnesemia, are associated with significant ingestion and were seen in the patient presented.

This case illustrates the importance of taking a complete medical history, including all non-prescription medications and supplements. Some patient supplements are obtained from internet sources, as was seen in this case, and a careful history is needed that goes beyond simply asking about current prescription medications. 
